# ﻿Four new erythroneurine leafhopper species from karst areas in Southwestern China (Hemiptera, Cicadellidae, Typhlocybinae, Erythroneurini)

**DOI:** 10.3897/zookeys.1204.122042

**Published:** 2024-06-03

**Authors:** Jinqiu Wang, Wenming Xu, Tianyi Pu, Ni Zhang, Yuehua Song

**Affiliations:** 1 School of Karst Science, Guizhou Normal University, Guizhou, Guiyang 550001, China Guizhou Normal University Guiyang China; 2 State Engineering Technology Institute for Karst Desertification Control, Guiyang 550001, China State Engineering Technology Institute for Karst Desertification Control Guiyang China

**Keywords:** Homoptera, morphology, new taxa, taxonomy

## Abstract

Four new erythroneurine leafhopper species, *Empoascanaraaparaoides* Wang & Song, **sp. nov.**, *Motagamengyangensis* Wang & Song, **sp. nov.**, *Motagaacicularis* Wang & Song, **sp. nov.**, and *Tautoneuraqingxiuensis* Wang & Song, **sp. nov.** from karst areas in Southwestern China, are described and illustrated.

## ﻿Introduction

Erythroneurini is the largest tribe of Typhlocybinae (Yuan et al. 2014). Erythroneurine leafhoppers are rich in diversity and have a body length of less than 5 mm. There are approximately 2,000 species worldwide, which are difficult to identify (Song and Li 2014). They feed on the leaf parenchyma cell contents and can cause harm to agricultural crops and forest trees of economic importance (Lu et al. 2021).

The genus *Empoascanara* was established by Distant (1918) with *Empoascanaraprima* Distant, 1918 as its type species. Subsequently, other researchers have described many new species. There are currently 92 *Empoascanara* species known, most of which are found in the Australian, Afrotropical, and Oriental regions. The genus *Motaga* was established by Dworakowska (1979) with *Motagarokfa* Dworakowska, 1979 as its type species. Only five species are known, and the genus is currently known only from the Oriental region. The genus *Tautoneura* was established by Anufriev (1969) with *Tautoneuratricolor* Anufriev, 1969 as its type species. It contains 64 species, of which 22 were previously known from China until now.

As part of this work, some interesting erythroneurine leafhopper materials from karst areas of Southwestern China were collected. Following examination and comparison of these materials, four new species, *Empoascanaraaparaoides* Wang & Song, sp. nov., *Motagamengyangensis* Wang & Song, sp. nov., *Motagaacicularis* Wang & Song, sp. nov., and *Tautoneuraqingxiuensis* Wang & Song, sp. nov., were discovered, and these are described and illustrated in this paper.

## ﻿Materials and methods

Specimens were collected by sweeping-net method. Male genitalia and abdominal apodemes were dissected and cleared in a 10% NaOH solution. Morphological terminology used in this study follows Dietrich (2005) and Song and Li (2013). The specimens were observed and drawn under Olympus SZX16 and Olympus BX53 microscopes, respectively. A Keyence VHX-5000 digital microscope was used for photography. The length of erythroneurine leafhoppers was measured from the apex of the head to the tip of the folded forewing. All specimens examined are deposited in the collection of the School of Karst Science, Guizhou Normal University, China (**GZNU**).

## ﻿Taxonomy

### ﻿ Empoascanara (Empoascanara)

Taxon classificationAnimaliaHemipteraCicadellidae

Distant, 1918

DDAF7427-7652-58A5-A838-626786CD948C

Empoascanara Distant, 1918: 94.

#### Type species.

*Empoascanaraprima* Distant, 1918, by original designation.

#### Description.

Dorsum yellow, white, pale red or brown. Crown broadly rounded medially. Vertex unicolorous, with a single dark median apical spot or a pair of spots. Crown nearly equal, slightly wider or narrower than widest part of pronotum. Pronotum pale, with darker posterior margin. Forewings with or without markings.

***Male genitalia*.** Pygofer microtrichia well developed. Pygofer lobe with caudal margin rounded or angulate. Dorsal pygofer appendage movably articulated, with or without ventral pygofer appendage. Subgenital plate expanded subbasally, with 2–4 basal macrosetae and numerous short stout setae along upper margin in lateral view. Style with preapical lobe prominent. Aedeagus with dorsal apodeme not or slightly expanded in lateral view. Aedeagal shaft usually symmetrical, slender in lateral view. Aedeagus with or without apical, subapical, or basal processes, and with or without preatrial ventral process or processes. Connective with median anterior lobe and arms short.

#### Distribution.

Oriental, Afrotropical, and Australian regions.

### ﻿ Empoascanara (Empoascanara) aparaoides

Taxon classificationAnimaliaHemipteraCicadellidae

Wang & Song sp. nov.

2600FA9E-C96D-50C1-9889-2E62C8DA8E94

https://zoobank.org/33509E60-DC24-4BDD-9B8E-6ECDA5536AEE

Figs 1–4 , 5–12

#### Diagnosis.

The new species can be distinguished from other species by the aedeagal shaft with one pair of longer subapical processes and one pair of shorter apical processes; the aedeagus without any basal process; the subgenital plate provided with three macrosetae on lateral surface; the pygofer dorsal appendage tapering towards apex; the connective with body strong, but lateral arms and central lobe short.

#### Description.

**Figures 1–4.**
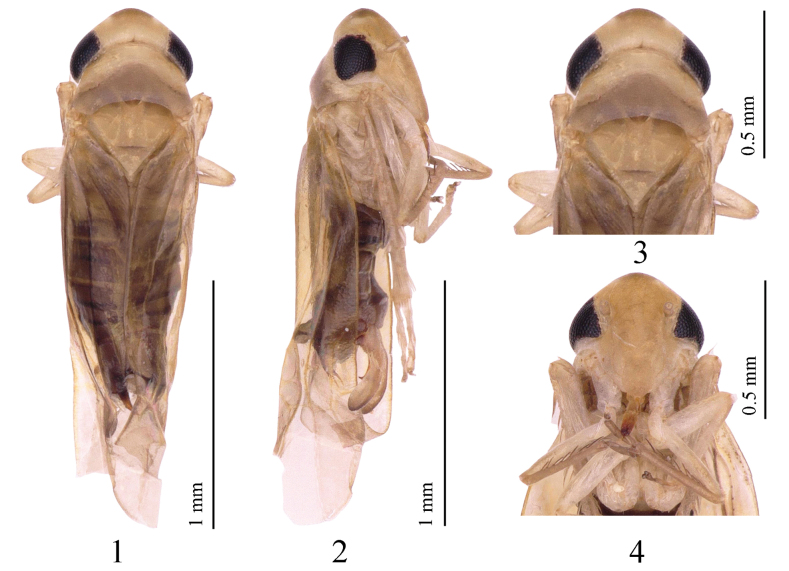
*Empoascanara (Empoascanara) aparaoides* Wang & Song, sp. nov. **1** habitus, dorsal view **2** habitus, lateral view **3** head and thorax, dorsal view **4** face.

Body small, ochraceous with brown markings. Vertex ochre-yellow; with one large, irregular, brown spot in middle of anterior margin (Figs 1, 3). Crown nearly equal to widest part of pronotum. Pronotum with anterior part ochraceous and brownish posterior part; posterior margin concave (Figs 1, 3). Crown with coronal suture short. Eyes black. Mesonotum ochraceous. Face milky yellow (Figs 2, 4). Forewing hyaline with brownish tinge (Figs 1, 3).

Male abdominal apodemes small, not exceeding 3^rd^ sternite (Fig. 12).

**Figures 5–12.**
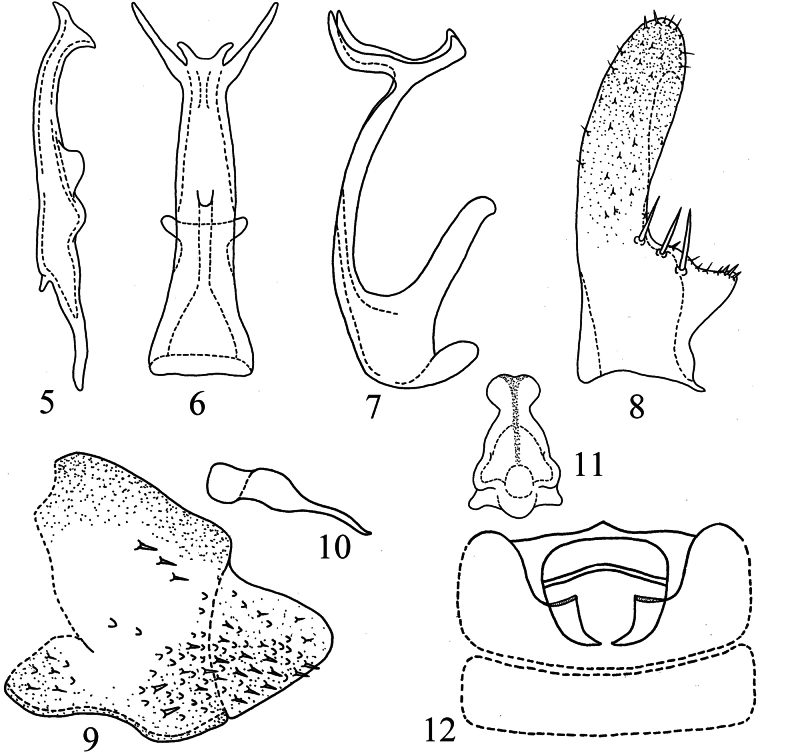
*Empoascanara (Empoascanara) aparaoides* Wang & Song, sp. nov. **5** style **6** aedeagus, ventral view **7** aedeagus, lateral view **8** subgenital plate **9** pygofer lobe **10** dorsal pygofer appendage, lateral view **11** connective **12** abdominal apodemes.

***Male genitalia*.** Pygofer lobe with numerous microsetae distributed densely at ventrolateral area and caudal part; three peg-like setae located on subdorsal area (Fig. 9). Dorsal pygofer appendage long, tapering towards apex (Fig. 10). Style slim (Fig. 5). Subgenital plate subbasally broadened, with three macrosetae on lateral surface, several peg-like setae distributed at subbase and apex; several microsetae scattered on apical part (Fig. 8). Aedeagal shaft long, provided with longer pair of subapical processes and a shorter apical pair of processes. Gonopore located at about mid-length of shaft on ventral surface (Figs 6, 7). Connective Y-shaped, with robust central lobe and two short lateral arms (Fig. 11).

#### Specimens examined.

***Holotype***: ♂; China, Yunnan Prov., Jinghong; 6 August 2021; Jinqiu Wang leg.; GZNU-2021-YN-JH-11-001. ***Paratypes***: 18 ♂♂, 24 ♀♀; same data as holotype; GZNU-2021-YN-JH-11-002 to 043.

#### Measurements.

Male length 2.3–2.4 mm, female length 2.4–2.5 mm.

#### Remarks.

This species is similar to *Empoascanaraapara* Dworakowska, 1979, but can be distinguished by its differently shaped pygofer dorsal process and an aedeagal shaft with one pair of long and one pair of short apical processes compared to only one pair of long processes in *E.apara*; also, the aedeagal shaft in *E.aparaoides* is without the medial hook-like process of *E.apara*.

#### Etymology.

The new species is named from the similar species, *E.apara*, the Greek suffix –*oides* denotes the similarity of the new species species to *E.apara*.

### ﻿ Motaga

Taxon classificationAnimaliaHemipteraCicadellidae

Dworakowska, 1979

1CB31BE9-4ECB-5BB7-BC68-A51BD7080465

Motaga Dworakowska, 1979: 12.

#### Type species.

*Motagarokfa* Dworakowska, 1979, by original designation.

#### Description.

Body gray to brown, without or with markings. Eyes gray to black. Crown fore margin weakly produced, broadly rounded apically. Pronotum usually without conspicuous pits. Mesonotum grayish brown. Forewing transparent or semitransparent. Peripheral vein at costal margin of hind wing absent.

***Male genitalia*.** Pygofer lobe broad, sparse setae on outer surface. Pygofer dorsal appendage curved ventrally in lateral view. Pygofer ventral appendage absent. Subgenital plate with 2–4 basal macrosetae; numerous short and stout setae forming continuous row from subbase to apex; several microsetae scattered on apical disc. Style apex truncated or expanded, foot-like. Connective with central lobe large. Aedeagus with dorsal apodeme expanded in lateral view; aedeagal shaft slender, curved dorsad in lateral view, with paired processes arising from base and shorter than shaft.

#### Distribution.

Oriental region.

### ﻿ Motaga mengyangensis

Taxon classificationAnimaliaHemipteraCicadellidae

Wang & Song sp. nov.

73B346BB-957F-5249-BF56-7B198D971C7A

https://zoobank.org/F4C1D37F-9AA3-4108-B407-937E4BCC7DBF

Figs 13–16 , 17–24

#### Diagnosis.

The new species can be distinguished from other species by the aedeagal shaft bifurcated at apex, crab claw-like, with one pair ½ length of aedeagal shaft basal processes; pygofer dorsal appendage expanded at base and tapering towards apex; subgenital plate with row of four macrosetae medially on outer surface; connective with central lobe broad and stem well developed.

#### Description.

**Figures 13–16.**
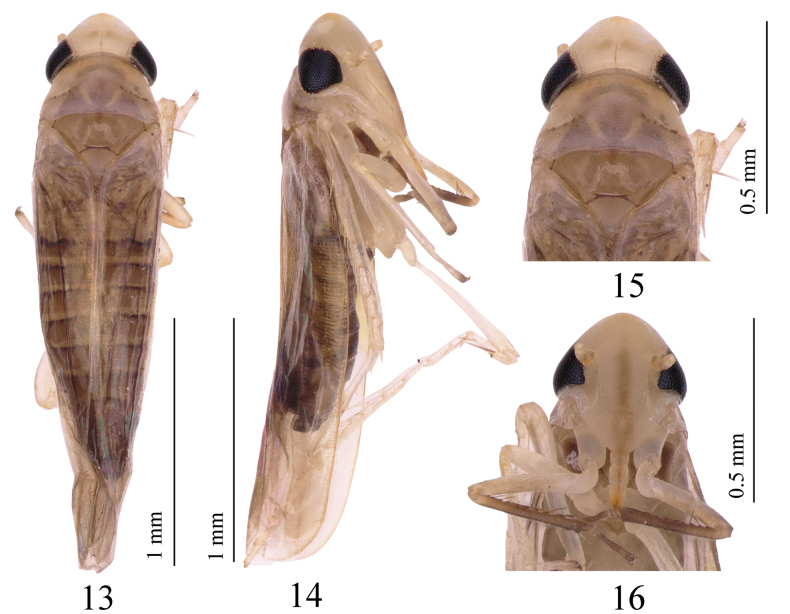
*Motagamengyangensis* Wang & Song, sp. nov. **13** habitus, dorsal view **14** habitus, lateral view **15** head and thorax, dorsal view **16** face.

Body brown (Figs 13, 16). Head slightly narrower than pronotum (Fig. 15). Crown fore margin strongly produced, with two irregular, medial, amber-colored patches (Figs 13, 15). Anterior part of pronotum light brown; posterior margin slightly darkened, with one nearly V-shaped, milky-white stripe. Coronal suture well developed. Eyes black (Figs 13, 14). Mesonotum brown. Forewing grayish brown (Fig. 14).

Male abdominal apodemes broad, extending to anterior margin of 4^th^ sternite (Fig. 24).

**Figures 17–24.**
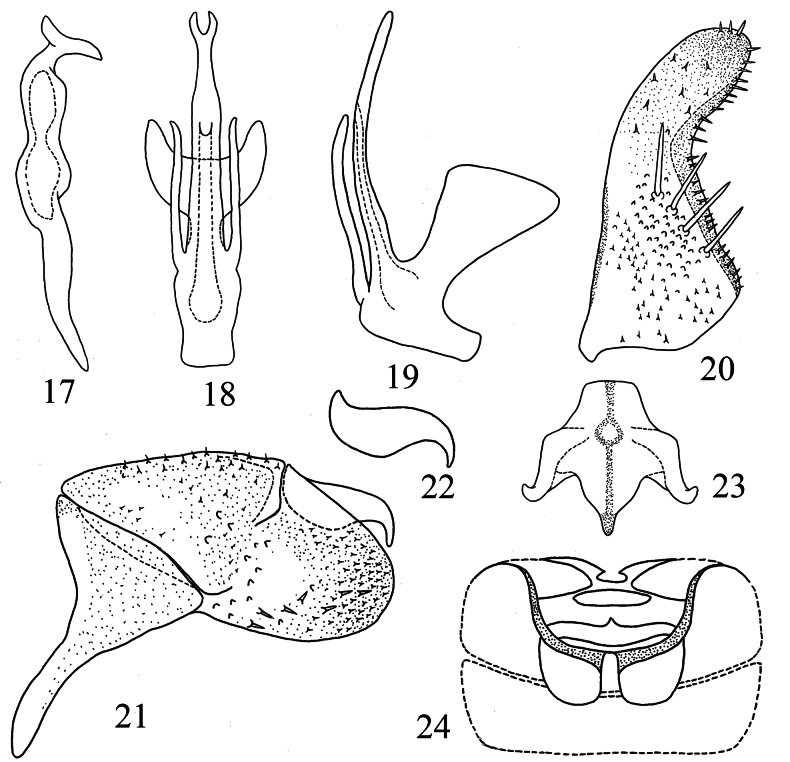
*Motagamengyangensis* Wang & Song, sp. nov. **17** style **18** aedeagus, ventral view **19** aedeagus, lateral view **20** subgenital plate **21** pygofer lobe **22** dorsal pygofer appendage, lateral view **23** connective **24** abdominal apodemes.

***Male genitalia*.** Pygofer lobe broad, with numerous microtrichia scattered along caudal edge and dorsal margin (Fig. 21). Dorsal pygofer appendage with wide base and sharp apex (Fig. 22). Subgenital plate with a row of four macrosetae in middle and with marginal peg-like setae from subbase to apex forming continuous row (Fig. 20). Style long and slender (Fig. 17). Connective with lateral arms strong, central lobe broad and stem well developed (Fig. 23). Aedeagal shaft long, straight in ventral view, curved dorsad in lateral view, bifurcated at apex; crab claw-like and with pair of basal long processes; gonopore located at 1/2 height of aedeagal shaft, ventrad (Figs 18, 19).

#### Specimens examined.

***Holotype***: ♂; China, Yunnan Prov., Jinghong City, Mnegyang Town; 2 August 2021; Tianyi Pu leg.; GZNU-2021-YN-JH-6-001. ***Paratypes***: 41 ♂♂, 58 ♀♀, same data as holotype; GZNU-2021-YN-JH-6-002 to 100.

#### Measurements.

Male length 2.3–2.4 mm, female length 2.4–2.5 mm (including wings).

#### Remarks.

This species is very similar to *Motagafara* Dworakowska, 1980, but it differs from *M.fara* in having the dorsal pygofer process with a stouter base, the length of the aedeagal shaft proportionally longer compared to the basal processes, and the gonopore located at about halfway along the length of the aedeagal shaft.

#### Etymology.

The new species is named after its type locality, Mengyang Town.

### ﻿ Motaga acicularis

Taxon classificationAnimaliaHemipteraCicadellidae

Wang & Song sp. nov.

5B08BA5E-D1A2-5C79-9B30-92022A876C0B

https://zoobank.org/4F5E5783-D358-4D31-82CE-C3E376DF6E64

Figs 25–28 , 29–36

#### Diagnosis.

The new species can be distinguished from other *Motaga* species by its extremely long and slender in lateral view aedeagal shaft, which has a pair of short basal processes that are not bifurcated at apex; the pygofer dorsal appendage, which tapers to the apex and is bent ventrad and hook-like apically; the connective with two long arms; the subgenital plate with four macrosetae; and the very small male abdominal apodemes.

#### Description.

**Figures 25–28.**
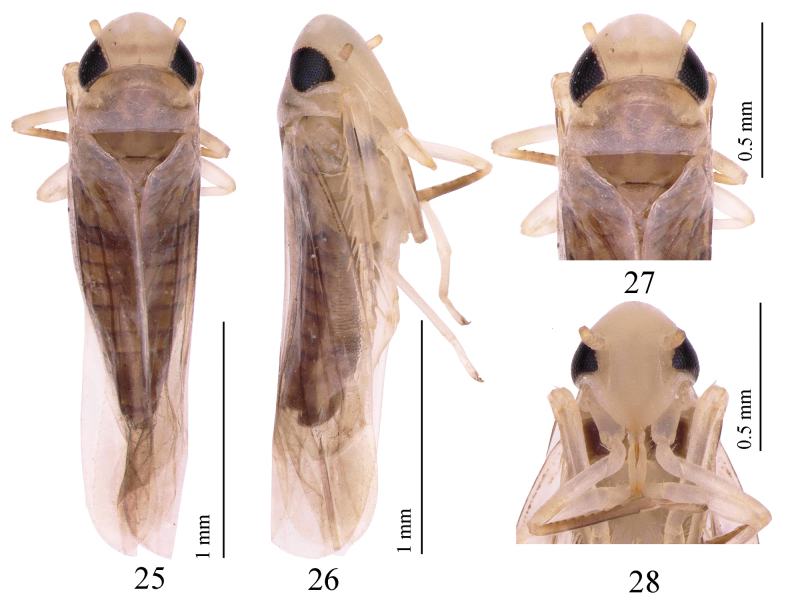
*Motagaacicularis* Wang & Song, sp. nov. **25** habitus, dorsal view **26** habitus, lateral view **27** head and thorax, dorsal view **28** face.

Vertex light brown (Figs 25, 27). Crown fore margin strongly produced, median length of crown slightly less than width between eyes (Figs 25, 27). Crown nearly equal to width of pronotum. Pronotum and mesonotum brownish yellow, posterior margin of pronotum almost straight (Figs 25, 27). Eyes black (Fig. 26). Forewings without spots, semitransparent (Figs 25, 26).

Male abdominal apodemes extremely small, not exceeding 3^rd^ sternite (Fig. 36).

**Figures 29–36.**
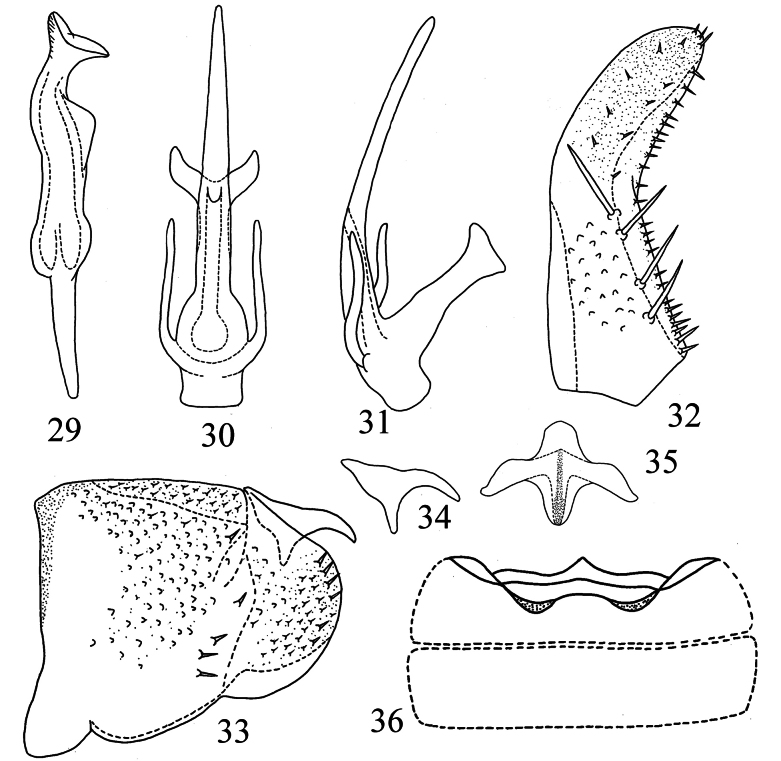
*Motagaacicularis* Wang & Song, sp. nov. **29** style **30** aedeagus, ventral view **31** aedeagus, lateral view **32** subgenital plate **33** pygofer lobe **34** dorsal pygofer appendage, lateral view **35** connective **36** abdominal apodemes.

***Male genitalia*.** Pygofer lobe broad, with numerous microtrichia; several peg-like setae scattered on middle area and hind edge (Fig. 33). Dorsal pygofer appendage with base expanded, with hook-like apex (Fig. 34). Subgenital plate with four macrosetae medially on lateral margin and numerous microsetae distributed along upper margin (Fig. 32). Style apex truncate and slightly expanded (Fig. 29). Connective with lateral arms robust, with obvious central lobe (Fig. 35). Aedeagal shaft long, slender, with paired processes at base (Figs 30, 31). Preatrium short; dorsal apodeme well developed, with apex bifurcate; gonopore located at basal 1/3 of aedeagal shaft (Figs 30, 31).

#### Specimens examined.

***Holotype***: ♂; China, Guangxi Prov., Liuzhou; 18 July 2021; Ni Zhang leg.; GZNU-2021-GX-LZ-8-001. ***Paratypes***: 96 ♂♂, 144 ♀♀; same data as holotype; GZNU-2021-GX-LZ-8-002 to 241.

#### Measurements.

Male length 2.3–2.4 mm, female length 2.4–2.5 mm (including wings).

#### Remarks.

This species is very similar to *Motagarokfa* Dworakowska, 1979 but can be distinguished by having the aedeagal shaft without a bifurcated apex, the preatrium expanded but short, and the paired basal processes approximately 1/3 length of aedeagal shaft.

#### Etymology.

The species epithet is the Latin word *acicularis*, which means slender, as a needle and refers to the needle-like aedeagal shaft.

### ﻿ Tautoneura

Taxon classificationAnimaliaHemipteraCicadellidae

Anufriev, 1969

F32A4D1A-4046-5C09-9F4D-DAA3966EA4F6

Tautoneura Anufriev, 1969: 186. Type species: Tautoneuratricolor Anufriev, 1969.
Erythroneura (Balila) Dworakowska, 1970: 347. Type species: Chloritamori Matsumura, 1906.
Havelia Ahmed, 1971: 277. Type species: Haveliaalba Ahmed, 1971.

#### Description.

Body white to yellow. Crown fore margin strongly produced medially, and slightly narrower or slightly wider than pronotum. Pronotum broad, with or without irregular spots. Mesonotum white to yellow, with basal triangles dark or indistinct. Forewing transparent, usually with single or multiple patches.

***Male genitalia*.** Pygofer lobe rounded, usually with several macrosetae at basal ventral angle and few peg-like setae at distal part on inner surface. Pygofer dorsal appendage slender and apically tapering, ventral appendage absent or present. Subgenital plate lateral margin distinctly widened subbasally, with 2–4 basal macrosetae. Style preapical lobe prominent, apex slender or truncate and expanded or with three points. Connective M- or Y-shaped, with slender median anterior lobe. Aedeagus dorsal apodeme usually expanded in lateral view; aedeagal shaft usually with single or paired processes apically and of variable length.

#### Distribution.

Palaearctic and Oriental regions.

### ﻿ Tautoneura qingxiuensis

Taxon classificationAnimaliaHemipteraCicadellidae

Wang & Song sp. nov.

5686249F-C943-58CA-B031-3ACD719ACB09

https://zoobank.org/F0B7D9BC-841C-4816-A9A2-0BD3B59BC2A7

Figs 37–40 , 41–48

#### Diagnosis.

The new species can be distinguished from other *Tautoneura* species by subapically broadened the aedeagal shaft in ventral view, with one pair of processes at apex; the extremely short preatrium; the apical gonopore; the dorsal pygofer appendage with base expanded; and the Y-shaped connective, with long, slim stem.

#### Description.

**Figures 37–40.**
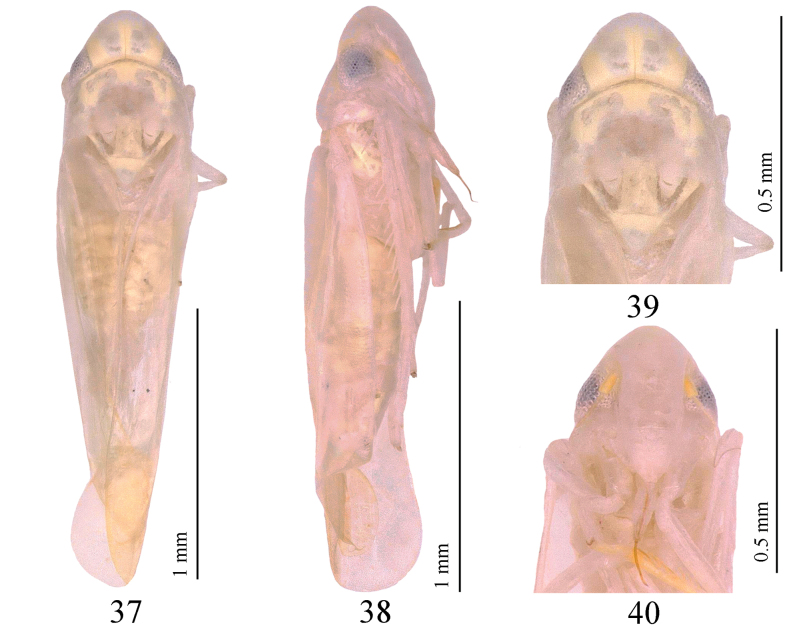
*Tautoneuraqingxiuensis* Wang & Song, sp. nov. **37** habitus, dorsal view **38** habitus, lateral view **39** head and thorax, dorsal view **40** face.

Body milky-yellow (Figs 37, 40). Vertex milky-yellow, with pair of gray-yellow spots on either side of coronal suture (Figs 37, 39). Crown yellowish, with fore margin strongly produced medially (Figs 37, 39). Eyes gray. Face milky-white, with base of antenna yellow (Fig. 40). Pronotum milky-yellow, with posterior margin whitish gray (Figs 37, 39). Mesonotum with basal triangles brownish yellow, but inside milky yellow (Fig. 39). Forewing transparent (Fig. 38).

Male abdominal apodemes narrow and extending to midlength of 4^th^ sternite (Fig. 48).

**Figures 41–48.**
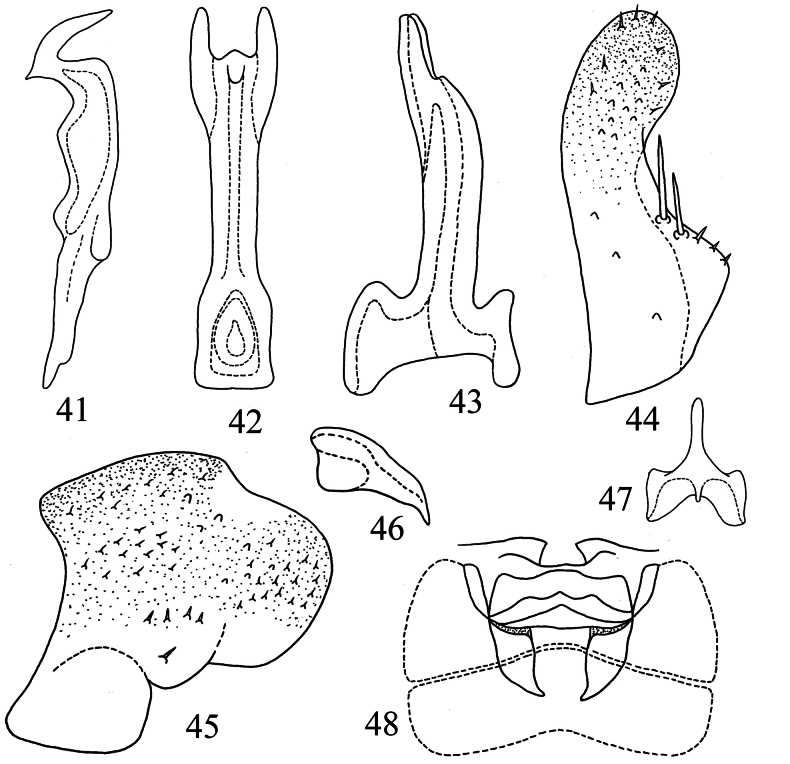
*Tautoneuraqingxiuensis* Wang & Song, sp. nov. **41** style **42** aedeagus, ventral view **43** aedeagus, lateral view **44** subgenital plate **45** pygofer lobe **46** dorsal pygofer appendage, lateral view **47** connective **48** abdominal apodemes.

***Male genitalia*.** Pygofer lobe with a few fine setae scattered on lateral surfac; pygofer microtrichia conspicuous, well developed; dorsal pygofer appendage distally tapered and basally expanded (Figs 45, 46). Subgenital plate with two macrosetae near middle aera of outer margin and some stout setae scattered near apex (Fig. 44). Style with prominent preapical lobe (Fig. 41). Connective Y-shaped, with long stem, two lateral arms strong, and central lobe rather small (Fig. 47). Aedeagal shaft nearly straight, subapically broadened in ventral view, with pair of processes at apex; gonopore near apex on ventral surface; dorsal apodeme slightly broadened in lateral view; preatrium extremely short (Figs 42, 43).

#### Specimens examined.

***Holotype***: ♂; China, Guangxi Prov., Nanning, Qingxiu Mountain; 21 July 2021; Wenming Xu leg.; GZNU-2021-GX-NN-3-001. ***Paratypes***: 2 ♂♂, 3 ♀♀; same data as holotype; GZNU-2021-GX-NN-3-002 to 006.

#### Measurements.

Male length 2.1–2.2 mm, female length 2.2–2.3 mm (including wings).

#### Remarks.

This species closely resembles *Tautoneuramaculosa* Sohi, Mann & Shenhmar, 1987, but it can be distinguished by the absence of prominent, dark markings on head, pronotum, and mesonotum (present in *T.maculosa*), the subgenital plate bearing two macrosetae (vs three), and the much stouter aedeagus than in *T.maculosa*.

#### Etymology.

The new species is named after its type locality, Qingxiu Mountain.

## Supplementary Material

XML Treatment for Empoascanara (Empoascanara)

XML Treatment for Empoascanara (Empoascanara) aparaoides

XML Treatment for Motaga

XML Treatment for Motaga mengyangensis

XML Treatment for Motaga acicularis

XML Treatment for Tautoneura

XML Treatment for Tautoneura qingxiuensis

## ﻿Additional information

### Author ORCIDs

Jinqiu Wang https://orcid.org/0000-0003-0675-8245

Wenming Xu https://orcid.org/0009-0004-8936-1650

Tianyi Pu https://orcid.org/0000-0002-6867-1527

Ni Zhang https://orcid.org/0000-0002-8604-8448

Yuehua Song https://orcid.org/0000-0003-3567-3056

### Data availability

All of the data that support the findings of this study are available in the main text.
